# First reports of nasal and traumatic myiasis infection in endangered Przewalski's horses (*Equus ferus przewalskii*)

**DOI:** 10.1016/j.ijppaw.2019.03.018

**Published:** 2019-03-24

**Authors:** Liping Yan, Ming Zhang, Liping Tang, Make Ente, Xinping Ma, Hongjun Chu, Kai Li, Defu Hu, Dong Zhang

**Affiliations:** a School of Nature Conservation, Beijing Forestry University, Beijing, China; b Xinjiang Research Centre for Breeding Przewalski's Horse, Xinjiang, China; c Wildlife Conservation Office of Altay Prefecture, Altay, Xinjiang, China

**Keywords:** Myiasis, China, Przewalski's horse, *Rhinoestrus*, *Wohlfahrtia magnifica*, Wildlife

## Abstract

Myiasis has great economic and medical importance. However, myiasis in wildlife that is caused by oestroid flies is relatively rarely recorded compared with that in humans and domestic animals. Recently, during our research on the conservation of Przewalski's horse (PH), we observed two new records of oestroid flies parasitizing wildlife in China. The first is the horse nasal bot fly, *Rhinoestrus* sp. (Diptera: Oestridae), found in a dead PH from Kalamaili Nature Reserve. One morphotype (*R. purpureus*-like) was identified. The second is the Wohlfahrt's wound myiasis fly, *Wohlfahrtia magnifica* (Schiner, 1862) (Diptera: Sarcophagidae), which was collected from an open wound of a PH in the Wild Horse Breeding Research Centre. These observations extend the records of known hosts of these two oestroid myiasis agents. To the knowledge of the authors, infestation by *Rhinoestrus* and *Wohlfahrtia* larvae causing myiasis in wildlife has not been reported in China previously.

## Highlights

• First report of *Rhinoestrus* sp*.* and *Wohlfahrtia magnifica* parasitizing Przewalski's horses.
• Infestation by *Rhinoestrus* and *Wohlfahrtia* larvae causing myiasis in wildlife has not been reported in China previously.
• This study will enrich the epidemiological data of myiasis infection of wildlife in China.

## Graphical abstract

**Figure:**
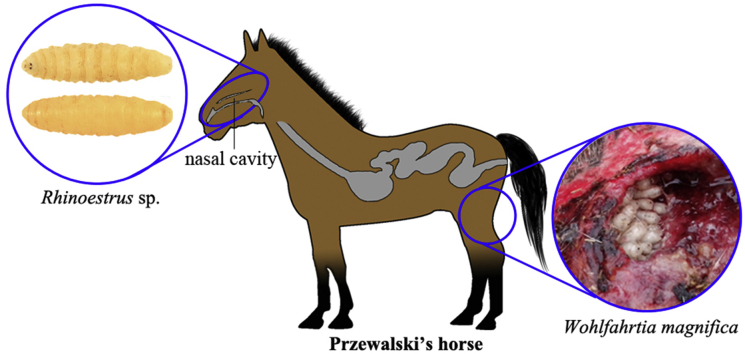

## 1 Introduction

Myiasis is the infestation of live vertebrates, including humans, with dipterous larvae, which at least briefly feed on dead or living tissue, liquid body substances, or ingested food of the host (Zumpt, 1965). The Oestridae, Calliphoridae, and Sarcophagidae represent the three major families of myiasis-producing flies (Hall and Wall, 1995). They all belong to the superfamily Oestroidea, making oestroid flies the primary myiasis agents. There are records worldwide of myiasis in people and domestic animals caused by oestroid flies (Smith et al., 2005; Gaglio et al., 2011; Singh and Singh, 2015; Hall et al., 2016), suggesting that they have considerable global economic and medical importance. Although myiasis is one of the most widespread parasitological problems in veterinary practices, the distribution and impacts of even the important agents are poorly recorded in wildlife compared with records of domestic animals, probably because of the high technical difficulty of obtaining such records for wildlife (Hall and Wall, 1995; Hall et al., 2016).

Our conservation research on Przewalski's horse (*Equus ferus przewalskii*, PH), the only extant wild horse species (Orlando et al., 2013), in Xinjiang over the past decade includes epidemiological data on stomach myiasis in equids and detailed biological information of horse stomach bot flies (Diptera: Oestridae, *Gasterophilus*) (Yang et al., 2013; Huang et al., 2016; Liu et al., 2016). Gastric myiasis caused by oestrids is very common in the released population from Kalamaili Nature Reserve (KNR) and the captive population from Xinjiang Research Centre for Breeding Przewalski's Horse (XRCBPH), especially in KNR, where almost all PHs are infested with *Gasterophilus* spp., resulting in gastric myiasis (Liu et al., 2016). The *Gasterophilus* parasite burden of deceased PHs in KNR can be more than 1000 individuals on average (Yang et al., 2013), which is much higher than the levels found in the Mongolian wild ass (*Equus hemionus hemionus*) and the local domestic horse (*Equus ferus caballus*) from the same area (Yang et al., 2013). A heavy parasitic load would risk the health condition of host and could even result in the death of the host (Czosnek, 1988; Getac hew et al., 2012). Therefore, we pay close attention to the myiasis in PH in order to conserve this iconic endangered species.

Unlike gastric myiasis, very little information is available regarding the nasal or traumatic myiasis in PH or other wild animals in China. The infection of traumatic myiasis occurs when fly larvae infest open wounds of a vertebrate host, accompanied by significant pain, swelling, irritation, or inflammation (Zumpt, 1965), leading to severe health problems (Hall et al., 2016). The nasal bots infest the nasopharyngeal cavities and internal organs of mammal hosts, inducing rhinitis, sinusitis, and even death of the host (Angulo-Valadez et al., 2010). Here, we present two new records of oestroid flies causing nasal and traumatic myiasis in PH, the horse nasal bot fly, *Rhinoestrus* sp. (Diptera: Oestridae), and the Wohlfahrt's wound myiasis fly, *Wohlfahrtia magnifica* (Schiner, 1862) (Diptera: Sarcophagidae).

## 2 Material and methods

### 2.1 Study area, specimen collection and identification

There are two re-introduced populations of endangered PHs in Xinjiang of China: the captive population in the XRCBPH located in Jimsar County (44°12′12″N, 88°44′26″E), with 89 individuals, and the free-ranging population in the KNR located in the southeast corner of the northeast Junggar Basin, with up to 130 individuals. The PHs described in the present study are under the general surveillance program of the Wildlife Conservation Office of Altay Prefecture, Forestry Department of Xinjiang. During May to June in 2014, one injured PH from XRCBPH and one dead PH from KNR were examined for myiasis infection (see section 2.2). All of the larvae recovered were either placed in a plastic cup to pupate or stored in 70% alcohol for subsequent identification in the lab. Species identification was carried out following morphological traits described by Zumpt (1965), Grunin (1966) and Colwell et al. (2006). Specimens were deposited in the Museum of Beijing Forestry University (MBFU), Beijing (Accession number: larvae of *Rhinoestrus* sp., BFU138-139 & BFU208-209; larvae of *W. magnifica*, BFU70-72; adults of *W. magnifica*, BFU205, BFU8710-8715).

This study was carried out in accordance with the guideline of the Institution of Animal Care and the Ethics Committee of Beijing Forestry University. All sample procedures in this study were performed with the help of a local veterinarian with approval of Wildlife Conservation Office of Altay Prefecture (Forestry Department of Xinjiang) and School of Nature Conservation (Beijing Forestry University).

### 2.2 Case descriptions

#### 2.2.1 Case I

A 10-year-old male PH at KNR was observed ill in the morning of May 2, 2014 and died about 4 h later. Two living third-instar dipteran larvae were found on the ground near its head. The larvae were assumed to be oestrids, so the horse's nasal cavity was probed with tweezers and another four living third-instar larvae were collected. Two of the larvae were brought to the lab to pupate, and the rest were stored in alcohol for morphological study.

#### 2.2.2 Case II

**Fig. 1:**
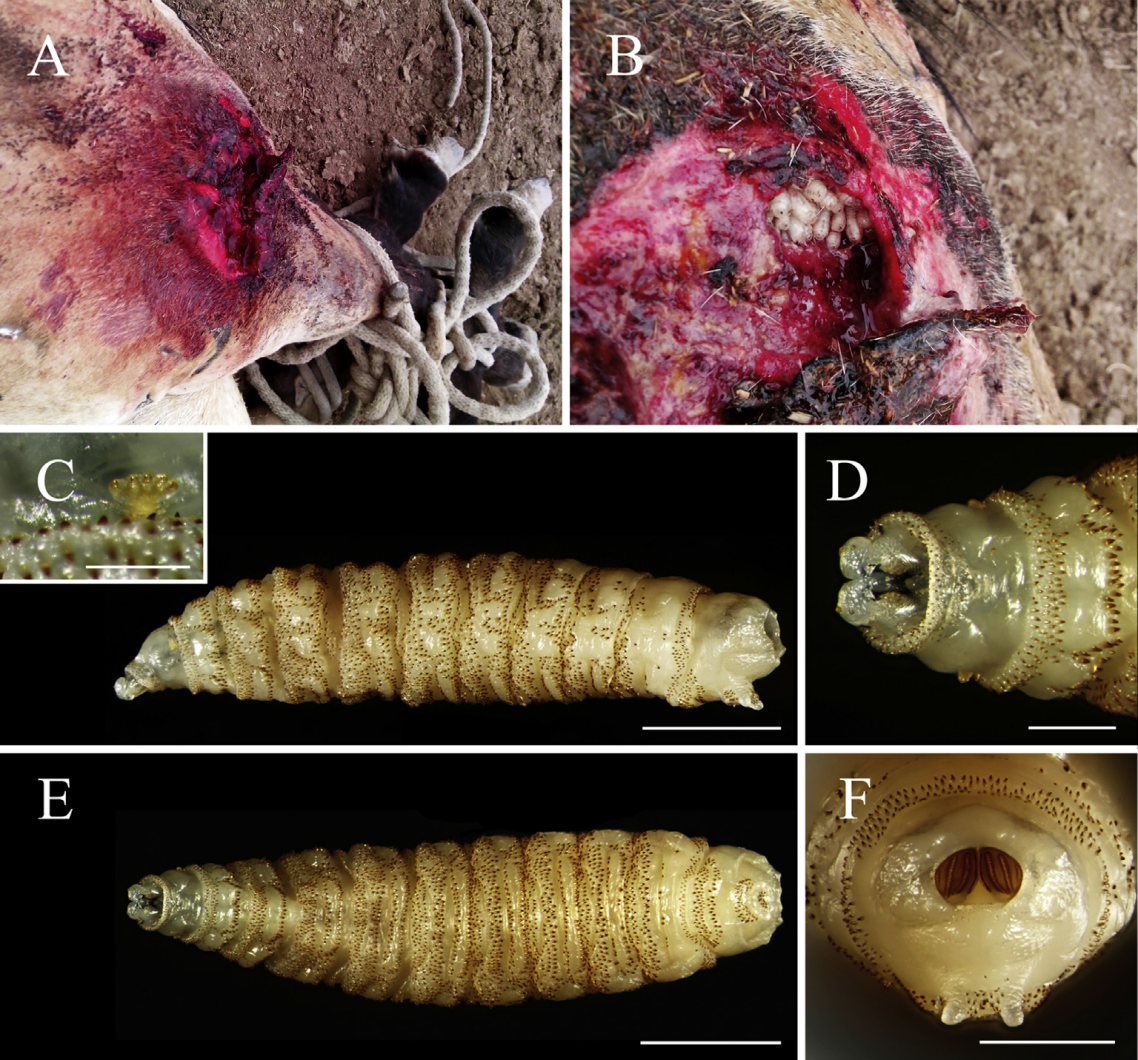
Photographs of the third larval stage of *Wohlfahrtia magnifica* (Schiner) in an injured Przewalski's horse in Xinjiang Research Centre for Breeding Przewalski's Horse, Xinjiang, China. A. The wound on the left stifle of the horse, restrained with rope. B. Wound after cleaning showing a cluster of larvae *in situ*. C. Lateral view. (Scale bar: 2 mm.) D. Ventral view of the anterior part. (Scale bar: 0.5 mm.) E. Ventral view. (Scale bar: 2 mm.) F. Posterior view. (Scale bar: 1 mm.)

An adult male PH raised at XRCBPH was hurt when fighting with another stallion on June 22, 2014, exposing a 10 cm × 10 cm wound on the left stifle (Fig. 1A). The PH was kept in a stall and could move freely. Three days later, it was immobilized to clean the wound, and dipteran larvae were found parasitizing the wound (Fig. 1B). A total of 52 larvae were collected. The wound was cleaned using normal saline before the PH was released. The PH has since recovered.

## 3 Results and discussion

**Fig. 2:**
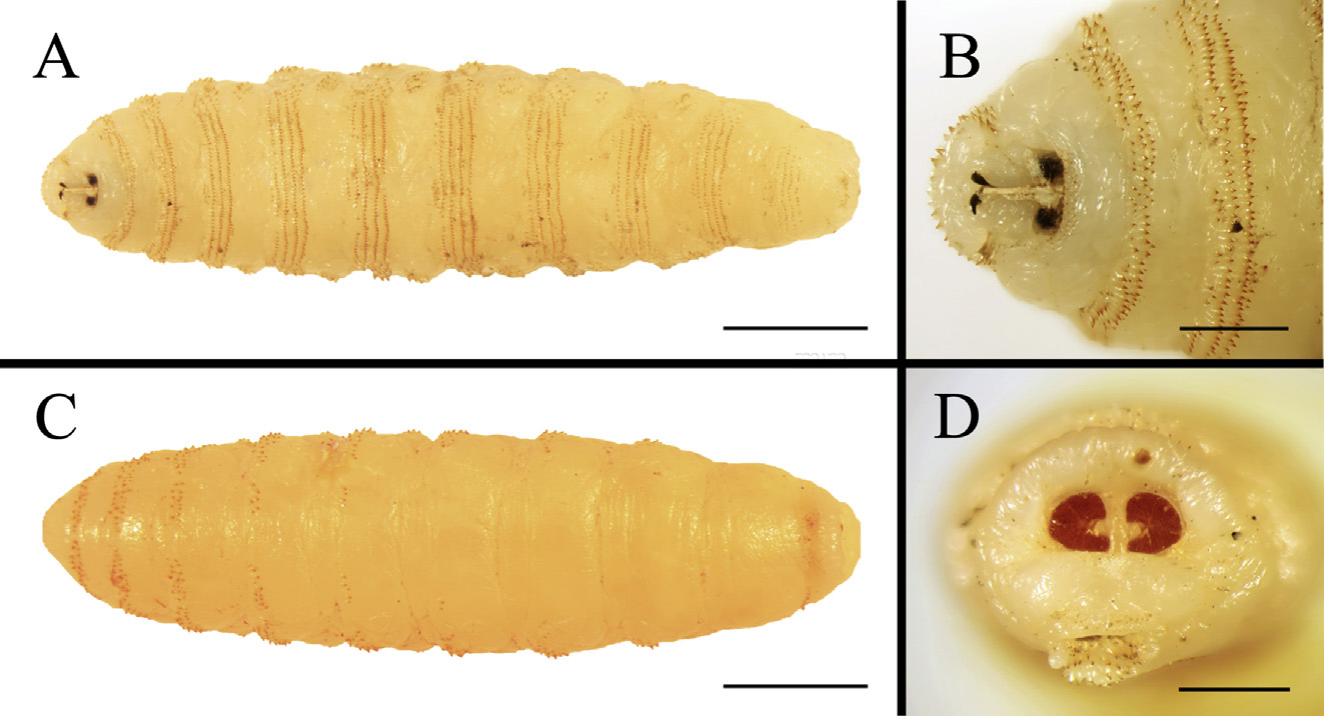
Photographs of the third larval stage of *Rhinoestrus purpureus-*like collected from Przewalski's horse in the Kalamaili Nature Reserve, Xinjiang, China. A. Ventral view. (Scale bar: 3 mm.) B. Ventral view of the anterior part. (Scale bar: 1 mm.) C. Dorsal view. (Scale bar: 3 mm.) Box showing the opening of the spines. (Scar bar: 0.25 mm.) D. Posterior view. (Scale bar: 1 mm.)

The larvae isolated from the nasal cavity of the PH in case I failed to pupate. One larva preserved in ethanol was identified as (*R*. *purpureus-*like) on the basis of the shape of the posterior spiracular plates (i.e., higher than broad) and the distribution of spines on the dorsal surface of the third segment (i.e., two complete rows of spines) (Fig. 2) (see Zumpt, 1965: Fig. 222; Otranto et al., 2005: Fig. 1B and C). Morphological characteristics used to identify *Rhinoestrus* spp. were mainly limited to the descriptions by Zumpt (1965) and Grunin (1966). However, morphological variability of *Rhinoestrus* spp. has been documented previously (Otranto et al., 2005; Mula et al., 2013; Hilali et al., 2015): these studies demonstrate that the morphological features used by Zumpt (1965) to separate *R. purpureus* and *R. usbekistanicus* affecting horses in Italy and Egypt were inadequate. Further studies are required to identify which *Rhinoestrus* species exist in China.

All larvae in case II (Fig. 1) were identified as *W. magnifica* according to the diagnosis of Zumpt (1965:109, Fig. 125), with 6, 25, and 21 individuals recognized to be first-, second-, and third-instar larvae, respectively. Fifteen of the third-instar larvae were brought to the lab, where they pupated on June 27th, and all emerged after 12 days. These two oestroid myiasis agents have not been previously reported in PHs in China.

*Wohlfahrtia magnifica*, one of the most important obligatory traumatic myiasis agents worldwide (Hall et al., 2016), is often found in domesticated animals, e.g., in wounds of dogs and horses (Diakakis et al., 2006; Farkas et al., 2009) and inside the vulva of goats (Gaglio et al., 2011). More than 200 larvae of *W. magnific*a can be found in a single host (Gaglio et al., 2011). Cases of humans infested with this species are also reported from time to time (Singh and Singh, 2015). In Xinjiang, the Wohlfahrt's wound myiasis fly was first recorded infesting humans in 1981 (Yao, 1981), and the existence of this species has been confirmed by occasional reports of infesting humans or domesticated animals and in subsequent investigations of dipteran diversity (Han et al., 1998; Tang et al., 2003). However, this is the first documented case of Wohlfahrt's wound myiasis fly parasitizing wild animals in China.

*Rhinoestrus purpureus* is an obligatory parasite of equids (Zumpt, 1965), and is rarely reported compared with Wohlfahrt's wound myiasis fly. Nevertheless, attacks on humans causing ophthalmomyasis and conjunctivitis by this species have been reported occasionally (Peyresblanques, 1964; Rastegaev, 1980). Myiasis caused by *Rhinoestrus* spp. in equids has rarely been reported in China and was confined to domestic Mongolian horses (Wang and Shi, 1963).

Although there is little information available concerning the impact of nasal and wound myiasis on wildlife in China, it is well known that myiasis is an important animal welfare problem, even if it rarely causes severe pathology (Hall and Wall, 1995; Colwell et al., 2006). Current control strategies, including insect light traps and applying regular chemical control, may be effective in decreasing populations of pest flies for captive host populations. However, long term strategies should pay close attention to predicting the distribution of pest species based on present distributions, since this may help determine where these two species occur currently and highlight where they may occur following dispersal or climate change (Hall et al., 2016).

## Funding

This study was supported by the Fundamental Research Funds for the Central Universities [No. JC2015-04], and the National Science Foundation of China [No. 31572305].
